# Does the mutual recognition agreement on nursing services accelerate nurse migration in member countries of the Association of Southeast Asian Nations?

**DOI:** 10.1002/nop2.504

**Published:** 2020-05-31

**Authors:** Sudo Kyoko, Kazuko Naruse, Boonyanurak Puangrat

**Affiliations:** ^1^ National College of Nursing National Center for Global Health and Medicine Tokyo Japan; ^2^ School of Nursing Tokyo Medical University Tokyo Japan; ^3^ Former Faculty of Nursing Thammasat University Bangkok Thailand

**Keywords:** Education, nursing workforce, qualitative approaches, quality of care

## Abstract

**Aim:**

To clarify the situations of nursing education and activity, its affecting factors and the nursing educators’ views on nurse migration relating Mutual Recognition Agreement on Nursing Services in the Association of Southeast Asian Nations. Design: Descriptive qualitative research.

**Methods:**

The individual semi‐structured interviews with 11 nursing educators, analysed using thematic analysis.

**Results:**

Nursing educators acknowledged that the change in nursing was mainly due to the creation and amendment of laws, acts and regulations regarding nursing and improvements in nursing education systems. Some of these improvements occurred by this mutual agreement. The conceptualization of the progress indicated an improvement in the quality of nursing. Nurse migration to the outside of Southeast Asian countries might be accelerated due to concurrent improvements in the quality of nursing. New trends among nurses working as caregivers in surrounding countries such as China, South Korea and Japan to deal with demographic ageing should be considered.

## 1 INTRODUCTION

Nurse migration began in the early 1940s. It became widespread during the 1990s when nurse shortages became a major issue due to rapid ageing worldwide and the ageing of nurses. Nurses migrated mainly from developing countries to developed countries for better wages (Chen et al., 2004). It has been pointed out that, in the 1990s, a widening gap in health care between source and destination countries (Stilwell et al., 2003) and unprofitable investments in nursing education due to the loss of strong visionary leaders in source countries (Buchan, 2001) arose as some negative aspects of nurse migration. Later, securing improvements in pay, working conditions, scheduling, career prospects and the security and prestige of nurses in their own countries were suggested (Buchan & Sochalski, 2004). Additionally, the following steps were proposed: encouraging and facilitating bilateral or multilateral flow management of nurses between countries and facilitating compensation flows from the recruiting country to the home country, the flow of remittances from nurses, educational support as part of a donor package, or the return flow of better‐trained staff (Buchan & Sochalski, 2004). Developed countries are still the main destinations for such nurses (Organisation for Economic Co‐operation & Development, 2018). The international rules for recruiting health professionals have been instated (World Health Organization, 2010). Therefore, nurse migration has developed within regional frameworks including the European Union (EU) and the Association of Southeast Asian Nations (ASEAN) or through bilateral agreements such as the Japanese Economic Partnership Agreement (JEPA).

The liberalization of goods and persons among the ASEAN countries has increased; many of these countries have concluded Mutual Recognition Agreements (MRA). Taxes on almost all goods were abolished with the establishment of the ASEAN Economic Community (AEC). The ASEAN MRA on Nursing Services (AMoNS) was formulated in 2006 (Association of Southeast Asian Nations, 2006) to support efforts to resolve the issues of rapid ageing and increasing socio‐economic gap among ASEAN countries (The ASEAN Joint Coordinating Committee on Nursing, 2016). Table 1 shows the objectives of AMoNS (Association of Southeast Asian Nations, 2006).

**TABLE 1:** Objectives of ASEAN Mutual Recognition Agreements on Nursing Services (Association of Southeast Asian Nations, 2006)

Object 1
Facilitate mobility of nursing professionals within ASEAN
Object 2
Exchange information and expertise on standards and qualifications
Object 3
Promote adoption of best practices on professional
Object 4
Provide opportunities for capacity building and training of nurses

The Eastern Asia and Pacific region is estimated to have the severest health worker shortage (Liu, Goryakin, Maeda, Bruckner, & Scheffler, 2017), mostly due to ageing. Japan, South Korea and China are experiencing extremely low birth rates and becoming super‐ageing societies (United Nations, 2019). This explains the rising healthcare needs among the neighbours of the ASEAN countries. Furthermore, Japan shares good relations with the ASEAN countries in several fields and is expected to contribute more towards ASEAN objectives. The Ministry of Health, Welfare and Labor of Japan has offered international contributions that are grounded in Japanese experiences of health policies for the elderly (Ministry of Health, Labour, & Welfare of Japan, 2014). Another factor affecting nurse migration in ASEAN countries is Japan's policy on care worker. The Japanese government has increased the opportunities for foreigners to work as “care workers” in Japan (Ministry of Health, Labour, & Welfare of Japan, 2019) despite the policy that basically the Japanese should be employed in the nursing care workforce in Japan (Ministry of Health, Labour, & Welfare of Japan, 2017b). Nurses and care workers from Indonesia, the Philippines and Vietnam have been accepted under the JEPA (Ministry of Health, Labour, & Welfare of Japan, 2017a). “Care worker” has been added as a status of residence in Japan for foreigners since 2017 (Ministry of Health, Labour, & Welfare of Japan, 2017b), as well as “Specified Skilled Worker (i)” since 2019 (Ministry of Foreign Affairs of Japan, 2019). In addition, the Technical Intern Training Program (TITP) for foreign care workers has also commenced since 2017 (Ministry of Justice & Ministry of Health, 2017). These occurred due to the urgent shortage of caregivers, especially for the elderly, as a result of decreasing population. Obtaining a job as a care worker in Japan is mainly based on two requirements. The first is adequate knowledge and skill in both the Japanese language and how to be a care worker in general. The second involves obtaining a certification as a care worker, by passing the national examination in Japanese to obtain permanent employment in Japan. Therefore, acquisition of basic nursing education is an advantage for foreigners desiring a care worker job in Japan. It could also be an incentive to migrate to Japan. Moreover, schools for care workers have not yet been established in most ASEAN countries. The number of certified care worker candidates accepted by JEPA increased dramatically as compared with the acceptance of nurses. In 2018, the number of nurse candidates was 97 and caregivers was 773, as compared with 112 for nurses and 109 for caregivers in 2013 (Japan International Corporation of Welfare Services, 2019). This is because the certified care worker candidates include graduates from nursing school in the home country. It has already been pointed out that well‐skilled Indonesian nurses are being “drained” to Japan as nurse aides or caregivers (Efendi, Chen, Nursalam, Indarwati, & Ulfiana, 2016; Kurniati, Chen, Efendi, & Ogawa, 2017). Its potential impacts should not be denied. This study focused on nurse migration, although the broader framework of health workforce including care workers should be considered.

The purpose of the study was to clarify the actual situations of nursing education and activity, the affecting factors in ASEAN countries and the ASEAN nursing educators’ views on nurse migration to verify whether AMoNS accelerated nurse migration among the ASEAN countries.

## 2 METHODS

We conducted a qualitative study based on individual semi‐structured interviews with 11 nursing educators from the ASEAN countries from March to October 2017 (see Table 2).

**TABLE 2:** Characteristics of the study participants (*N* = 11)

ID	Nationality	Current position in home country	Age
1	Cambodia	Nursing lecturer	32
2	Cambodia	Government officer	47
3	Indonesia	Nursing lecturer	42
4	Indonesia	Nursing lecturer	26
5	Lao PDR	Nursing lecturer	50
6	Myanmar	Nursing lecturer	41
7	Myanmar	Nursing lecturer	42
8	The Philippines	Nursing lecturer	37
9	Vietnam	Nursing lecturer	35
10	Vietnam	Nursing lecturer	35
11	Vietnam	Nursing lecturer	37

The participants were students of a graduate course in Thailand with nursing work experience and basic nursing education from their own countries. We targeted the ASEAN nurses from the supposed source countries. The largest number of migrants in Thailand came from the ASEAN countries. Thailand was a leader in nursing education not only in the ASEAN region but also around the world, and the Thai government had accepted international students from the ASEAN countries, especially Cambodia, Lao PDR and Myanmar. Indonesia and the Philippines also concluded the JEPA. Moreover, we supposed that nurses studying in the graduate course would take leadership roles and have a strong interest in improving nursing education and practice at home. This study was approved by the Ethical Review Committee of the National Center for Global Health and Medicine; the approval number was NCGM‐G‐002158–00.

The researchers contacted the participants through the Thai researcher, and their permission and informed consent were individually sought. Japanese researchers interviewed the ASEAN nurses while referring to an interview guide and recorded the interviews with a recorder. All participants have provided written consent for publication of the data collected.

The original interviews were conducted with reference to the factors of nurse migration (Buchan, Prkin, & Schalski, 2003), AMoNS objectives (Association of Southeast Asian Nations, 2006) and achievements (The ASEAN Joint Coordinating Committee on Nursing, 2016). The questions regarded actual situations of nursing education and activity, influential factors including AMoNS in each country and the ASEAN nursing educators’ views on nurse migration.

We used content analysis (Krippendorff, 2004) along with MAXQDA Plus12 for all analytical processes (VERBI Software GmbH, 2015). We created a transcript based on the recorded interviews and extracted meaningful sentences fitting the study objectives based on syntactical distinction and thematic distinctions. These were categorized by similarity to understand, interpret or relate to the intended decisions easily. Categories were defined as mutually exclusive and exhaustive using verbal designation and decision schemes. The reliability of the results was determined based on their stability and reproducibility; multiple analysts worked under varying conditions at different locations to ensure reproducibility. The validity was confirmed by selecting the sample based on plural subjects of interest. The consensus among skilled researchers regarding repeated discussions helped exclude arbitrariness.

## 3 RESULTS

Data from the interviews were categorized into five themes: “Laws, Acts and Regulations”; “Nursing education system”; “Quality of nursing in clinical settings”; “Effect of ASEAN Mutual recognition on Nursing Services”; and “Factors affecting nurse migration.” The themes and represented codes extracted are shown in Table 3.

**TABLE 3:** Themes and contents extracted

Theme (number of codes)	Represented codes
Laws, Acts, and Regulations (8)	Amending Act and regulations regarding nursing and midwifery Establishing rules and regulations for nursing by the nursing and midwifery council Not existing accreditation system for nurses Not existing licensing examination Operating new regulation for all health professionals Preparing regulation to respect patients Stipulating the regulation of all professional practice
Nursing education system (116)	Accrediting nursing schools with a diploma course and a lecturer by government evaluation Improving the curriculum of nursing including nurse competency, nursing process, and nursing ethics Improving the educational skills of nursing teachers using new teaching methods Improving quality of nursing teachers to be sent to other countries by government support Increasing the number of nursing universities providing bachelor's degrees Lacking opportunities for nursing educators to upgrade themselves, with continued professional development Lacking advanced nursing education, Masters, and PhD Providing information on nursing legal concepts, nursing management and leadership, and ethics Requiring the nursing research skill of a lecturer by the government
Quality of nursing in clinical settings (78)	Improving the professionalism of nurses Incurring the differences of nurses as professional by their education level Conducting training and programs involving nursing leadership and management in all clinical settings by the Nursing Association Demotivating to improve because of the doctor's perspective of them as an assistant Existing unwelcome nurses’ allocation system by the government Improving the management skill of hospital nurses upgraded to the bachelor's degree through the bridging course Introducing the nursing process to nurses in a clinical setting Lacking decision making by nurses themselves, following the medical doctor's decision Lacking ethical consideration and legal concept, knowledge and skills of leadership and management, research skills, and theoretical thinking as a nurse professional in a clinical setting Lack of human resources Not renewing the knowledge and skill of nurses in a public hospital Requiring certification to be a head nurse
Effect of ASEAN mutual recognition on nursing services (64)	Accepting foreign nurses Applying as a foreign nurse to the Philippines and Malaysia which use English Being in progress to implement MRA Opening the door to work and study abroad Desiring of nursing students to study abroad Desiring to work abroad without adequate competence Conducting a nursing student exchange program Giving a chance to improve research together among ASEAN member countries Improving the curriculum of nursing to adopt world standards Improving nursing education to meet the criteria of ASEAN standards Not accelerating nurse migration even after MRA Not recognizing MRA in all nursing settings Preparing an exchange program between foreign countries by the government Sending nursing teachers for training to Thailand following MRA requirement Understanding among ASEAN member countries
Factors affecting nurse migration (50)	Basic education before entering nursing school Language barrier Concluding bilateral agreement with a developed country Improving international relationships Improving the quality of nursing according to a patients' needs Improving technology Lacking budget to adopt MRA requirements Lacking nurses in a hospital Freely migrating to a developed country Nurses’ consideration of cultural diversity Policy on population control Political power Restriction by government to work abroad Support for and acceptance of international students from developing countries by universities in another country

The nursing educators who were interviewed recognized that situational changes in nursing in recent decades had resulted mainly from amendments to nursing laws, acts and regulations and also from improvements in the nursing education system. This finding corresponded to the facts gathered from the document review, which assured that implementation had taken place. However, they were sceptical about the improvement in the quality of nursing in clinical settings. They believed that the effect of AMoNS had generated some improvements. Although AMoNS stimulated the intention to study or work abroad, other factors also affected nurse migration.

This section contains excerpts from the interview transcripts modified to improve grammar. Excerpts are integrated into paragraphs below for matters of flow and are indicated with italics.

### 3.1 Laws, acts and regulations

Relevant laws existed in each country and had been amended to fit AMoNS requirements:

In 2004, we had only one national nursing policy…later on, we developed a lot of documents like Code of Ethics, Standard of Nursing Practice and Nursing and Midwifery Procedures. (Cambodia)

We have had this nursing law since 1994. In 2002, it was revised to consider a nurse as a professional. (Philippines)

The Ministry of Health implements new regulations now to improve communication with patients… and every hospital has to change its way of approaching patients. (Vietnam)

### 3.2 Nursing education system

All of the participants recognized the importance of upgrading basic nursing education:

In the 1990s, our nurses were almost at secondary level… from 2000 until now, our government upgraded nursing schools to colleges and universities [and] recently, the Ministry of Health has made a new regulation to stop recruiting the nurses who were educated within the two‐year curriculum system by 2021. (Vietnam)

We have more nurses who have bachelor’s degrees and the number is increasing. (Lao PDR)

Simultaneously, the universities increased their authority:

Our University has its own authority to collaborate with affiliated teachmaing hospitals under the control of the Ministry of Health (Myanmar)

Participants also recognized that the curriculum had been improved to cultivate professionalism, such as nursing leadership and management, ethics and legal concepts and nursing competency. A course for nurse specialists was started:

We must teach ethics even in diploma curriculums. (Cambodia)

We discuss the different concepts at the hospital in the leadership and management course…as students could assume managerial roles when they become nurses in clinical settings. (Philippines)

Nurses in emergency, ICU, medicine, surgery, pediatrics, ICU pediatrics and anesthetics… we can call them professional nurses [leaning] about becoming a nursing specialist for 4‐6 months as part of their bachelor’s degree course. (Lao PDR)

New teaching methods were introduced. The equipment and materials for teaching were also changed:

The active participation of students in lecturers and group discussion has improved and is encouraged… audiovisuals or teaching aids in the skills lab are adequately provided [so that] students can learn with visual data or other simulated materials. (Myanmar)

They implement new teaching methods such as case studies, more interactions and critical thinking. (Vietnam)

Every university has an online system; universities are creating new websites to provide information. (Indonesia).

Nursing educators perceived the necessity of further improving nursing education:

One of the reasons why I came to Thailand is to study for my PhD; in my country, we only have three schools that offer PhD courses. (Philippines)

Nursing teachers only teach students; they do not upgrade their own knowledge…very few can attend conferences or seminars. (Cambodia)

Students who are more clever [than others] will study in the government universities, but private universities do not have high standards; there is a huge discrepancy between the two. (Indonesia)

### 3.3 The quality of nursing in clinical settings

The quality of nursing activity seemed to improve, but nursing educators felt that the next step required more consideration. They implied that improvements in nursing quality in clinical settings proceeded at a slower pace than in nursing education:

The quality of nursing is changing and creating a good impact…being a nurse professional, it is necessary to continue learning. (Lao PDR)

We are struggling for autonomy, but it is much better than before. Other professionals also appreciate the importance of nursing, care techniques, collaboration skills and management. (Myanmar)

Before, we only were functionally a team. Now, we have become able to work as a team. (Cambodia)

The nurse’s perception is based and depends on the doctor’s decision; we need more time to emphasize this…The doctor‐to‐nurse ratio in Vietnam is very low, so we have asked the government for more nurse positions. (Vietnam)

In our regulations, nurses are required to have at least a bachelor’s degree in nursing,…But a nurse must possess good and moral values in accordance with the standards and the ethics of nursing practice, to be considered a nursing professional. (Philippines)

We have to know our responsibility and our own as well as the patients’ rights under law,…but nurses don’t want to know them, though they face many troubles due to their lack of awareness. (Indonesia)

### 3.4 The effect of ASEAN mutual recognition on nursing services (AMoNS)

Nursing educators believed that nursing education had improved due to AMoNS:

The university has to accredit nursing educators to meet the ASEAN standards [and] try to produce higher level nurses who meet those criteria. (Myanmar)

I think [we are] now moving toward improving the curriculum [because] a nurse who is trained in the Philippines now has to meet the needs of not only Philippine society but also the world. (Philippines)

Other significant effects of AMoNS were growing recognition and acceptance of nurse professionals among the ASEAN countries and the emerging motivation and preference to work abroad. Consequently, improvements were implemented in the situation:

Every year, we sit together with nurse leaders from different countries…so I know that they know the situation in Cambodia very well. (Cambodia)

From this MRA, our government recognized the position of nurses in the healthcare system and put in more efforts to send nurses to study abroad or to invite international organizations. I think this is a very good chance or a trigger to improve nursing. (Vietnam)

Training outside leads to improvement in nurses… We are a low‐income country, so it is good for us if nurses can work abroad. (Lao PDR)

We are trying to send our students to Indonesia and other countries…. It has a kind of relation to MRA, I think. (Myanmar)

The Philippines is known as a market for nurses all over the world a long time before this issue came up…I think that now the nursing labor market is becoming more open to other countries like here in Thailand. (Philippines)

However, AMoNS did not seem to accelerate nurse migration within ASEAN or other countries:

Nurses go to Thailand and Singapore but not to Malaysia; some go to Japan for a short term. If they are allowed by the government, it is easy, but usually there is no support, so nurse migration is limited. (Lao PDR)

MRA was concluded with ten nations, but it is very difficult for our nurses to move to other countries for work. (Vietnam)

Even if the door will open for ASEAN countries, I think nurses will have to consider the currency [for] they will not opt to work in that country if the currency is worth a lower value compared to the Philippines’ currency. (Philippines)

### 3.5 Factors affecting nurse migration

According to nursing educators, the factors affecting nurse migration were basic nursing education, the education system before entering nursing school, financial limits, language barriers, national policy, other agreements and people's demands to study or work abroad:

Earlier, elementary education was for six years and high school was four years—so 10 years in total—but now it has changed to 12 years. The curriculum has been revised again because of this impact of compulsory education” (Philippines)

We don’t have the budget to implement MRA activities. (Cambodia)

Nobody wants to go, as their English isn’t good” (Lao PDR)

In 2006, they signed the MRA, but we didn’t know about it. The new government has taken power and changed a lot of things since 2015. (Myanmar)

We signed a Memorandum of Understanding as a government agreement with two nations, Japan and Germany. (Vietnam)

People in my country are really willing to go abroad to get a job with high pay if they can, not only people in the nursing profession. (Cambodia)

## 4 DISCUSSION

### 4.1 Improvements in nursing due to ASEAN mutual recognition on nursing services

There have been changes in nursing education and practice since 2006, as can be seen in the report “AJCCN Achievements (2007–2015)” (The ASEAN Joint Coordinating Committee on Nursing, 2016). According to nursing educators, the main change in the last decade was the establishment or amendment of laws and regulations regarding nursing, especially the nursing regulation or examination system established in Lao People's Democratic Republic (PDR) and Vietnam (Fujita et al., 2019; Sonoda et al., 2017). This finding suggested that such laws and acts or the introduction of new regulations regarding nursing led to improved nursing education, as shown by the upgrading of nursing education systems. Notably, all countries that participated in this study had experienced both.

However, the implementation of improvement in clinical settings has been slow and this issue has not been discussed in the report (The ASEAN Joint Coordinating Committee on Nursing, 2016). Patient‐centred care, protection of patients’ and healthcare workers’ human rights and the pursuit of professionalism, which nursing educators demonstrated in this study, are necessary for improving nursing quality. AMoNS is expected to contribute towards improving nursing quality because each ASEAN country has made efforts to comply with the regulations. The result in the theme “the quality of nursing in clinical settings” showed changes in the professionalism of nurses, led to the conduction of leadership and management training, as well as imbibing the importance of having a common concept of the nurse profession. These results indicate ASEAN countries’ attempt to follow the regulations established or revised after AMoNS. Moreover, the difference in nurses’ professionalism based on their education level and the knowledge gap between nursing students studying in the current curriculum and nurses in a clinical setting indicates the need to improve the quality of nursing in clinical settings in the near future.

On the other hand, the increasing motivation to work abroad has also indirectly led to improvements in the quality of nursing. Opportunities to work as a nurse abroad such as the country, which has a working environment requiring expertise and skill and patients’ high demand for disciplined interaction with health workers, may increase once the quality of nursing improves.

Unexpectedly, ASEAN countries’ recognition and acceptance of each other emerged in the analysis as one of the positive aspects of AMoNS. It suggests that AMoNS contributes to the increasing transparency in ASEAN countries to help them understand each other. This mutual understanding also leads to the improvement of the quality of nursing.

### 4.2 The relationship between nursing quality and nurse migration

Nurse migration within the ASEAN region was premised on the standards of each ASEAN country according to AMoNS. This situation under AMoNS is different from the situation in the European Union under EU regulations. In the EU, which has cultural diversity, free mobilization for nurses is also allowed and member countries provide special programmes such as language support and “a career ladder” to support migrant nurses (Keighley, 2009; Sumption, Papademetriou, & Flamm, 2013; Zander, Blumel, & Busse, 2013). Generally, international nurse migrations rarely occur if there is a significant gap in the quality of skill and knowledge between countries and there is no monetary incentive for migration (Asadi et al., 2017; Bidwell et al., 2014; Santric‐Milicevic et al., 2015). Nursing educators concurred with this view based on their observations of actual ASEAN‐related migration situations. A differential in the progress of AMoNS and an unresolved initial gap that we found during the interviewing may explain the acceleration of nurse migration within the ASEAN region.

**FIGURE 1:**
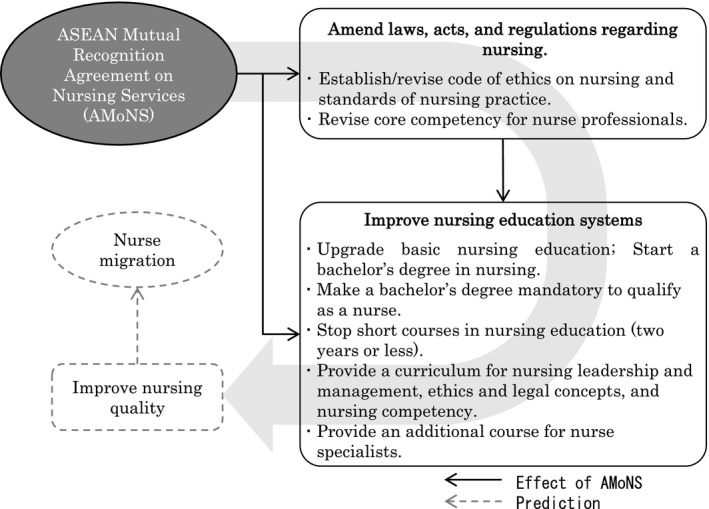
The progress of ASEAN Mutual Recognition Agreement on Nursing Services

We attempted to conceptualize the progress of AMoNS by reviewing other documents and the interviews. As shown in Figure 1, the presence of the AMoNS effect might have improved nursing quality. This suggests that nursing quality led nurse migration to accelerate in accordance with the final goal of AMoNS. Nurse migration to countries outside the ASEAN region under other agreements and conventional nurse migration to other developed countries may accelerate in future owing to the improvements in the quality of nursing.

### 4.3 Nurse migration trends in the ASEAN countries

International movement of unskilled/low‐skilled workers from Cambodia, Lao PDR and Myanmar to Malaysia, Thailand and Singapore has been occurring because of various pull and push factors within the ASEAN region (Sugiyarto & Agunias, 2014). Nevertheless, nurse migration within the ASEAN region still faces challenges (Hofmeister, Rueppel, Pascouau, & Frontini, 2014; Matsuno, 2009); the reasons presented in this study matched those of previous studies, such as wide economic gap, individual health policies, diverse views of nursing professionalism and nursing authority.

The results indicate that AMoNS only helped facilitate nursing professionals’ mobility within the ASEAN region. However, the results also show that improvements in nursing quality due to AMoNS might accelerate nurse migration further. Migration to countries outside the ASEAN region should be considered because destination countries that have other agreements with ASEAN countries (e.g. Germany and Japan) have a huge demand for elder‐care workers.

### 4.4 Limitations

Participant selection was one limitation of this study. Nursing educators who are also international students made the criteria for participant narrow. However, they are more likely to describe their countries’ situations accurately because the nursing educator studying in other countries can explain both nursing education and nursing activity in the clinical setting in their home country with wide view comparing with other countries. The other limitation was that the number of participants from each ASEAN country was limited. This was also caused by participant selection. The participants who meet the study requirements could also be recruited from universities in other countries such as the United States of America, Australia or Japan, although they participated only in Thailand.

## 5 CONCLUSIONS

Since 2006, laws, acts and regulations regarding nursing and nursing education systems in the ASEAN countries have improved dramatically. However, nursing activities in clinical settings have improved at a slower pace. Nursing educators have not perceived AMoNS’ contribution towards nurse migration despite a decade of lapse. This relevant that the quality of nursing after concluding basic regulation regarding nursing and improving nursing education results in nurse migration. And the study indicates its process is in progress.

Once the quality of nursing in the ASEAN countries improves in the future due to AMoNS, the strong demand from other countries experiencing a rising need for caregivers for the elderly may accelerate nurse migration. Several aspects of nurse migration should be considered for the further improvement of nursing quality. Additionally, discussions among policymakers and nurse leaders on new nurse migration trends and a general willingness of nurses from ASEAN countries to work as caregivers or “care workers” are essential.

## CONFLICT OF INTEREST

No conflict of interests has been declared by the authors.

## AUTHORS’ CONTRIBUTION

KS and KN conceived the study design and carried out data collection with a substantial contribution from PB. KS and KN performed the analysis and drafted the manuscript, and PB conducted critical revision. All authors reviewed and approved the final manuscript.
